# Architecture‐Controlled Hierarchical Carbon‐Based Current Collector for Mitigating Interfacial Instabilities in Lithium‐Metal Anodes

**DOI:** 10.1002/advs.75769

**Published:** 2026-05-22

**Authors:** Seo Hui Kang, Dong Hyeon Hwa, Ji Su Chae, Kwang Chul Roh

**Affiliations:** ^1^ Climate and Energy Research Group Korea Institute of Ceramic Engineering and Technology Jinju Gyeongsangnam‐do Republic of Korea; ^2^ Space & Energy Technology Team Innovation Technology Research Division Korea Research Institute for Defense Technology Planning and Advancement Daejeon Republic of Korea; ^3^ Department of Ceramic Engineering School of Materials Science and Engineering College of Engineering Gyeongsang National University Jinju Gyeongsangnam‐do Republic of Korea

**Keywords:** anode‐free battery, carbon‐based current collector, Li host site, Li metal battery, three‐dimensional current collector

## Abstract

The growing demand for lightweight and high‐energy‐density storage systems has renewed interest in lithium metal batteries. Despite this interest, their commercialization is still constrained by challenges such as dendritic Li growth and parasitic interfacial reactions. In this study, an architecture‐controlled hierarchically porous carbon‐based current collector consisting of carbon nanotubes and mildly steam‐activated carbon black is proposed, in which controlled defects in the carbon black provide lithiophilic nucleation sites while causing minimal degradation of its electronic conductivity. This lightweight and mechanically robust architecture (0.69 mg cm^−^^2^) ensures efficient electron transport and promotes spatially uniform Li‐ion flux, thereby confining lithium nucleation within the porous matrix and effectively suppressing dendrite formation and interfacial instabilities at the Li‐metal interface. Electrochemical characterization reveals that the current collector retains 50.4% of its initial capacity at 2C, maintains 77.6% of its original capacity over extended cycling, and achieves an average Coulombic efficiency of 96.2%. Additionally, scanning electron microscopy analysis confirms that Li nucleation predominantly occurs within the internal pore network rather than on the external surface. These findings demonstrate that the proposed architecture‐controlled carbon current collector offers a scalable strategy for stabilizing Li‐metal anodes in safe, high‐energy‐density rechargeable batteries for applications in electric mobility and aerospace systems.

## Graphical abstract

A defect‐engineered, hierarchically porous carbon black/CNT current collector guides lithiophilic nucleation in 3D architecture. Steam‐created defects enable uniform Li plating, while CNTs provide conductivity and a lightweight framework, suppressing dendrites and side reactions for stable anode‐free cells.

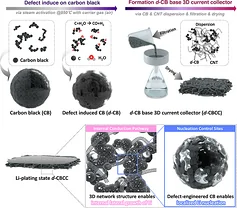

## 1 Introduction

With the increasing demand for high‐performance energy storage devices, lithium‐ion batteries, sodium‐ion batteries, supercapacitors, and various electrochemical systems have been widely studied [1, 2, 3, 4]. Advances in material design and interface engineering have led to notable improvements in energy density, rate capability, and cycle stability. These developments are particularly important for applications such as electric vehicles and large‐scale energy storage systems, where high energy density is required.

Among these systems, lithium metal batteries have attracted considerable attention as next‐generation energy storage devices due to their high theoretical capacity (3,860 mAh g^−1^) and low electrochemical potential (−3.04 V vs. standard hydrogen electrode) [5]. In addition, the inherently low atomic weight of lithium contributes to enhanced gravimetric and volumetric energy densities by maximizing the proportion of active material [6]. The growing demand for lightweight and high‐energy‐density systems is particularly relevant for applications such as electric vehicles and aerospace platforms [7, 8].

Despite these advantages, their practical application remains limited. Although significant research efforts have been devoted to improving lithium metal batteries, challenges associated with non‐uniform lithium deposition and dendritic growth still persist. These issues can lead to safety concerns, unstable electrode–electrolyte interfaces, and poor cycling stability, ultimately hindering their practical implementation.

To reduce the inactive mass, recent studies have focused on anode‐free Li metal batteries (AFLMBs), wherein Li is directly deposited onto a bare current collector without the use of excess Li metal [9, 10]. AFLMBs offer the benefits of simplifying cell fabrication and enabling high energy density. However, despite continuous progress in electrode material development, conventional copper metal base current collectors (CCs) still account for a considerable portion of the anode mass (∼25%) while contributing little to the overall capacity [10]. Replacing these dense and electrochemically inactive foils with lightweight, highly conductive, and lithiophilic alternatives is therefore expected to enhance both the gravimetric and volumetric energy densities, which is particularly advantageous in aerospace applications where weight constraints are critical. In addition, the formation of electrically isolated “dead Li” during repeated cycling is considered a key factor contributing to irreversible capacity loss and limited long‐term stability in AFLMBs. This issue mainly arises from non‐uniform Li nucleation and dendritic growth, which can lead to the electrical isolation of deposited Li from the current collector. As a result, the accumulation of inactive lithium gradually reduces the amount of electrochemically active Li and destabilizes the electrode–electrolyte interface [11, 12, 13].

For the development of next‐generation Li metal batteries, the CCs must meet critical requirements, including uniform Li nucleation, effective dendrite suppression, high electronic conductivity, and minimal parasitic mass. To meet these stringent requirements, current collector designs have evolved along three principal strategic directions [14, 15, 16]. Firstly, surface‐engineered metal foils coated with lithiophilic oxides, fluorides, or ultrathin nitrides via atomic layer deposition or electrochemical conversion techniques are effective in lowering the nucleation overpotential [17, 18]. However, such additional coatings increase process complexity and do not fundamentally mitigate the mass penalty associated with high‐density metallic substrates. Secondly, three‐dimensional (3D) metallic architectures fabricated by Cu/Ni foaming, extrusion‐based printing, or laser structuring techniques have been shown to facilitate spatially uniform Li deposition by increasing the surface area and extending the internal pore networks. Nevertheless, their intrinsic high densities and sluggish ion transport through tortuous metal pathways restrict any improvements in the gravimetric energy density [19, 20]. Thirdly, carbon‐based CCs derived from oxidative, acidic, or plasma treatments of graphitic frameworks have been widely explored to introduce nanoscale defects that serve as Li nucleation sites [21, 22]. However, when applied aggressively, such harsh chemical or plasma routes often disrupt the sp^2^‐hybridized carbon lattice and generate a high density of oxygenated groups, which can significantly reduce electrical conductivity and impair the mechanical robustness of the carbon host [23]. As a result, it remains challenging for these defect‐engineered carbon CCs to simultaneously satisfy the requirements of controlled architecture, interfacial stability, and lightweight design in Li metal batteries [21].

We introduce a lightweight hierarchical carbon current collector (*d*‐CBCC) based on steam‐activated carbon black integrated with carbon nanotubes, designed to guide confined lithium nucleation and deliver superior electrochemical performance. That is fabricated via a purely physical steam activation process that enables the precise tuning of both nanoscale porosity and surface defect density while preserving the underlying graphitic framework. Subsequently, the activated carbon black is uniformly integrated with CNTs to form a freestanding, binder‐free carbon scaffold with a well‐defined hierarchical architecture. This hierarchical architecture induces an internal confinement effect, directing Li nucleation and deposition uniformly within the porous matrix and thereby effectively mitigating dendritic growth and interfacial instabilities at the Li‐metal anode. Furthermore, the interconnected CNT network facilitates continuous and efficient electron‐transport pathways throughout the electrode. Compared with conventional metallic foils or chemically modified carbon‐based CCs, the proposed CC exhibits significantly lower areal mass loading, enhanced lithiophilicity, and superior electrochemical cycling stability. This architecture‐controlled design strategy therefore offers a promising route to lightweight, high‐performance of CCs in high‐energy‐density Li metal batteries by enabling the coupled modulation of nano‐porosity and surface chemistry while maintaining mechanical and structural robustness.

## 2 Results and Discussion

**SCHEME 1:**
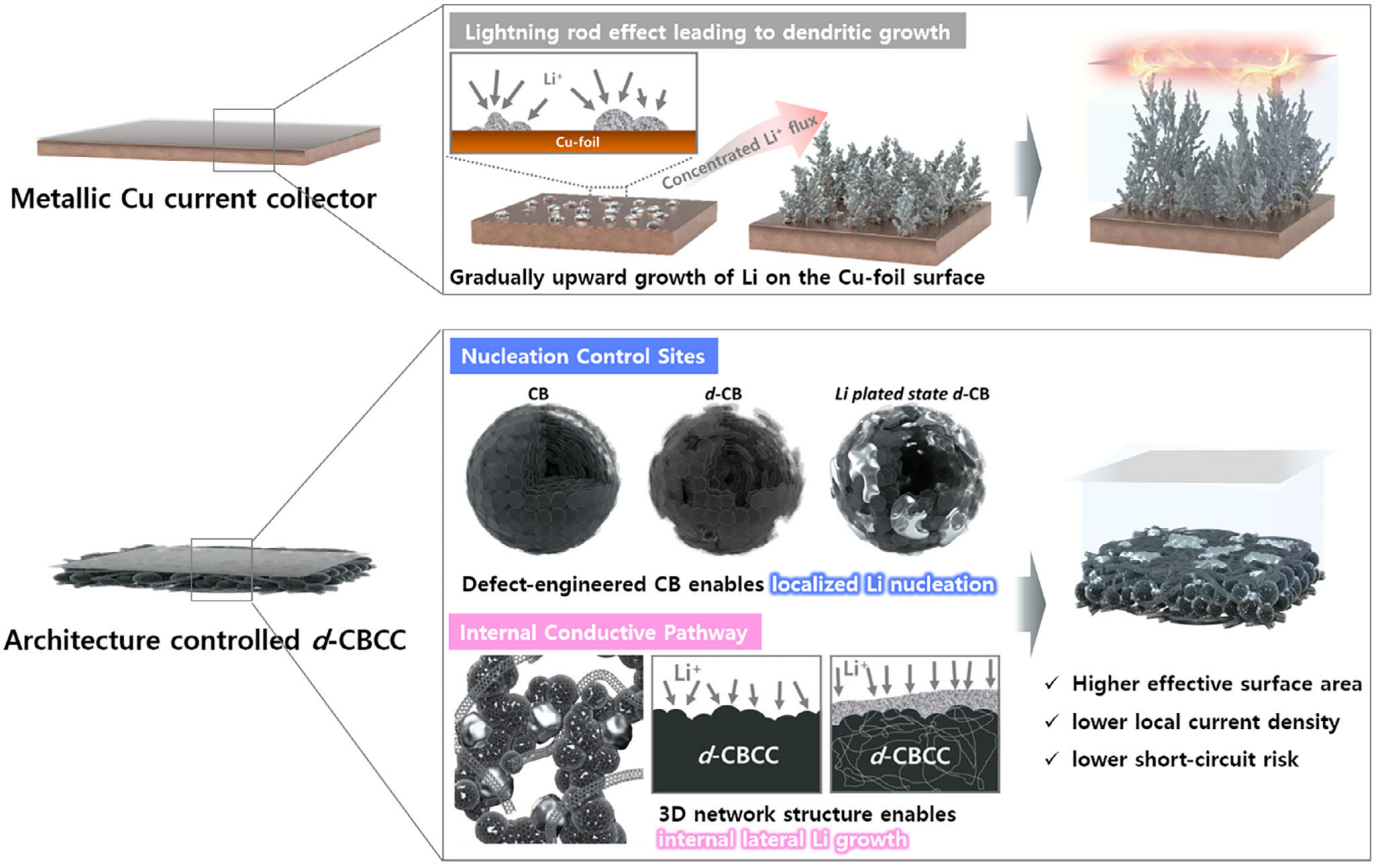
Comparison of an architecture‐controlled *d*‐CBCC and a conventional metallic Cu CC for use as an anode material in lithium metal batteries.

Scheme 1 compares Li deposition and stripping on a bare Cu CC with that on a defect‐tailored 3D CNT‐based CC to clarify how the architecture of the CC governs the deposition mechanism. Since Cu is a non‐intercalation substrate, Li plates directly onto its surface under galvanostatic conditions. Repeated Li^+^ shuttling creates local current‐density heterogeneities that promote the formation of mossy, high‐surface‐area Li‐deposits, which are characteristic of intrinsically hostless Li‐metal [24]. These deposits disrupt the solid electrolyte interphase (SEI), leading to the accumulation of electrically isolated “dead” Li that can ultimately penetrate the polymeric separator. The CNT collector mitigates these failure modes through the combined effects of its percolating, high‐surface‐area conductive network, along with its defect‐tailored chemistry. Additionally, its 3D architecture increases the electrochemically active surface area, homogenizes ion flux and interfacial current distribution, and reduces both the effective areal current density and the local overpotential [25]. Consequently, nucleation and growth are biased toward the scaffold interior, and tip‐driven dendritic growth is suppressed, while void formation is mitigated during the stripping process. In parallel, carefully introduced defect sites in the carbon structure, including vacancies, edge planes, and mild heteroatom dopants, provide lithiophilic sites that lower the Li nucleation overpotential and yield interconnected, laterally continuous deposits embedded within the framework [21, 22]. Such coupling between the material structure and its inherent chemistry delivers lower nucleation overpotentials, a more robust and less reactive SEI, improved reversibility, and higher Coulombic efficiency during long‐term cycling, which translates to an enhanced safety profile relative to that of bare Cu. However, the benefits associated with such systems depend on the route of defect introduction, since benign and predominantly physical treatments preserve graphitic conductivity, whereas harsh oxidation introduces excessive oxygen functionalities that promote parasitic electrolyte decomposition and gas evolution, ultimately degrading electronic conductivity.

**FIGURE 1:**
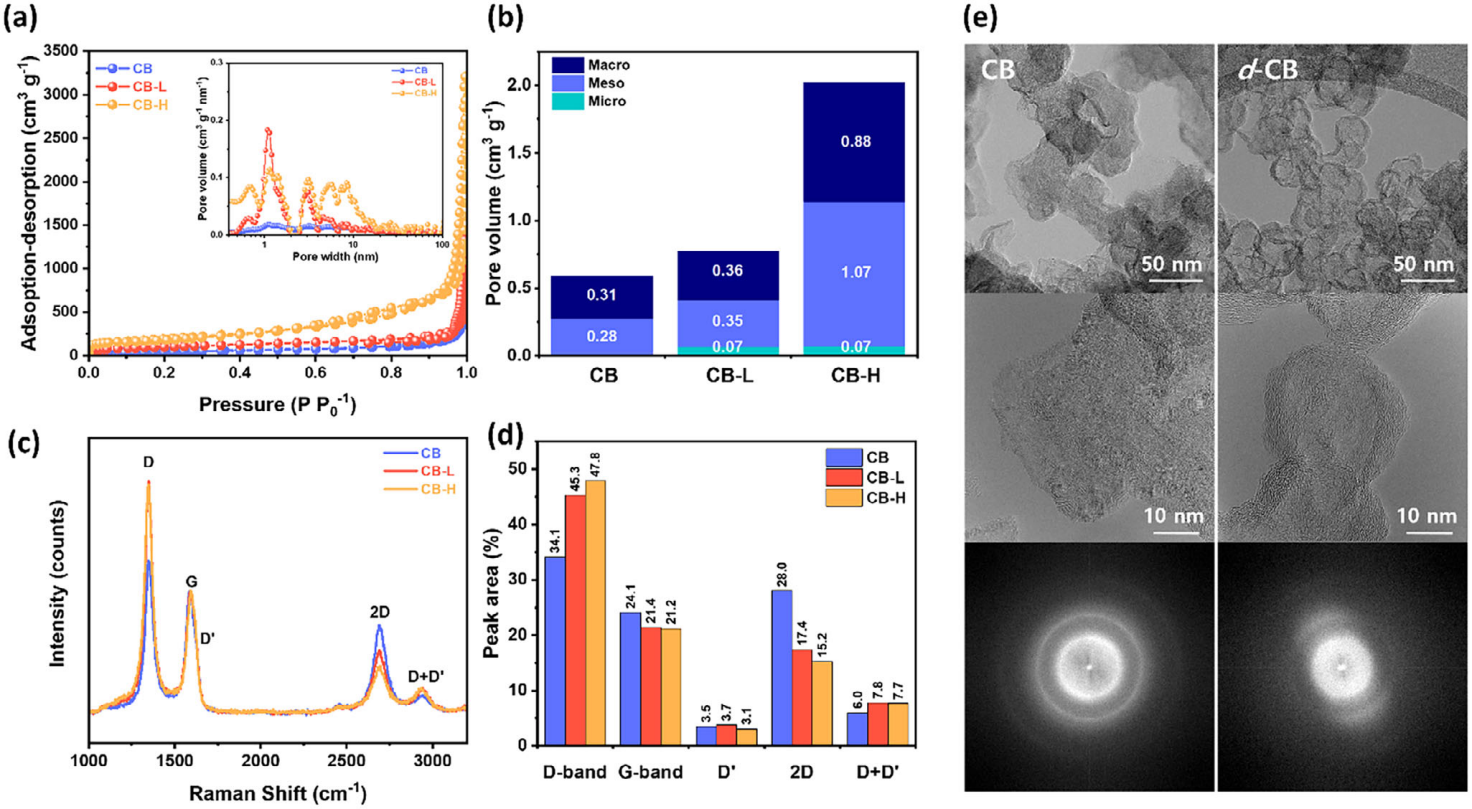
Structural characterization of the pristine CB powder and steam‐activated powder samples (CB‐L and CB‐H). (a) N_2_ adsorption–desorption isotherms (type IV with a hysteresis loop). The inset shows the pore‐size distributions derived using the NLDFT model. (b) Pore volume distributions as a function of the pore diameter, evidencing the development of hierarchical micro–mesoporosity upon steam activation. (c) Raman spectra. (d) Deconvoluted Raman band area fractions, indicating a progressive increase in the defect density with the activation severity (i.e., higher *I*_D_/*I*_G_ ratios). (e) Cs‐TEM images and corresponding SAED patterns of CB and *d*‐CB with a higher defect density, revealing steam activation‐induced lattice distortion and reduced crystallinity.

The pore structures of the pristine CB and the steam‐activated derivatives with low (CB‐L) and high (CB‐H) defect densities were investigated using N_2_ adsorption–desorption isotherms (Figure 1a). All three materials display Type IV isotherms accompanied by H3 hysteresis loops, typical of slit‐shaped mesoporous networks. In the pristine CB, the minimal uptake in the low relative pressure range (P/P_0_ ≤0.01) confirms the scarcity of micropores. In contrast, as the activation duration increases, the total adsorption rises progressively, signifying pore enlargement along with the development of additional mesoporous (2–50 nm) and macroporous (>50 nm) domains. For the CB‐H specimen, a steep uptake is observed near saturation (P/P_0_ ≈ 1.0), indicating pronounced macropore growth. Similar tendencies are captured in the non‐local density functional theory (NLDFT)‐derived pore size distributions (Figure 1a, inset), which clearly demonstrate the emergence of a hierarchical porous network containing micro‐, meso‐, and macropores following prolonged activation [26].

The pore volume partitioning results (Figure 1b) further emphasize these structural variations. Specifically, the micropore volumes remain essentially constant across the samples, whereas the mesopore and macropore fractions increase sharply. For the CB‐H specimen, the mesopore and macropore volumes reach 1.07 and 0.88 cm^3^ g^−1^, respectively, substantially exceeding those of the pristine CB. This transformation is consistent with the steam activation process (Equation (1), (2), (3), (4)), where localized oxidation at the exposed surfaces initiates micropore formation; these micropores subsequently merge and expand to generate mesopores and macropores [27].

(1) $$\mathrm{Adsorption}\;\mathrm{at}\;\mathrm{the}\;\mathrm{active}\;\mathrm{site}:\;C\;\left(s\right)+H_{2}O\;\left(g\right)\;\to \;C\cdot H_{2}O_{2}\;\left(s\right)$$

(2) $$\mathrm{Water}\;\mathrm{homolysis}:\;H_{2}O\;\left(g\right)\;\to \cdot \mathrm{OH}\;(g)+\cdot H_{2}\;\left(g\right)$$

(3) $$\mathrm{Hydrogen}\;\mathrm{formation}:\;C\cdot \mathrm{OH}\;(s)+\cdot H\;(g)\;\to \;{\mathrm{CO}}_{2}\;\left(g\right)+H_{2}\;\left(g\right)$$

(4) COdesorption:C·O(s)→CO(g)

Raman spectroscopy (Figure 1c) reveals a steady increase in the *I*_D_/*I*_G_ ratio upon prolonging the activation time, confirming the progressive generation and spread of defects within the carbon lattice [28, 29]. This indicates that steam activation promotes defect density formation at both the surface and within the bulk, representing a microstructural evolution process that can potentially impact the overall electrochemical performance.

Deconvolution of the Raman spectra (Figure 1d and Figure S1) highlights the growing prominence of defect‐associated bands, such as the D' and D + D' bands, upon increasing the activation time. These features point to the gradual formation and enlargement of edge‐plane terminations and vacancy‐type defects, which increase the prevalence of electron‐deficient domains. Such domains are known to lower the nucleation overpotential for Li‐metal deposition, promoting more uniform nucleation and enhancing the lithiophilic nature of the carbon matrix.

The x‐ray diffractometry (XRD) results presented in Figure S2 corroborate these findings. Specifically, upon prolonging activation, the intensity of the graphitic (002) reflection diminishes, suggesting partial degradation of long‐range crystalline ordering. However, CB‐L retains higher crystallinity than CB‐H, indicating that lower‐defect activation conditions better preserve graphitic stacking. Together, these analyses provide quantitative and qualitative evidence for defect generation and microstructural transformation driven by steam activation, highlighting a strong correlation with the mechanisms responsible for enhanced Li deposition in subsequent electrochemical tests.

Cs‐corrected transmission electron microscopy (TEM) imaging (Figure 1e) confirms the occurrence of nanoscale morphological changes. Although all samples consist of agglomerated spherical primary particles measuring ∼30–50 nm in diameter, for the defect‐induced CB (*d*‐CB) system, the mean particle size is reduced, and the graphitic fringes appear more distinct. For CB‐H, these fringes are shorter and discontinuous, indicating increased amorphization. The selected area electron diffraction (SAED) patterns support this interpretation, wherein CB‐H shows diffuse diffraction rings characteristic of short‐range disorder, while the pristine CB exhibits sharper rings consistent with preserved long‐range order. This microstructural and defect evolution is expected to improve electrolyte infiltration into the current collector and ensure a more uniform Li^+^ flux, thereby helping to suppress dendritic growth during cycling.

**FIGURE 2:**
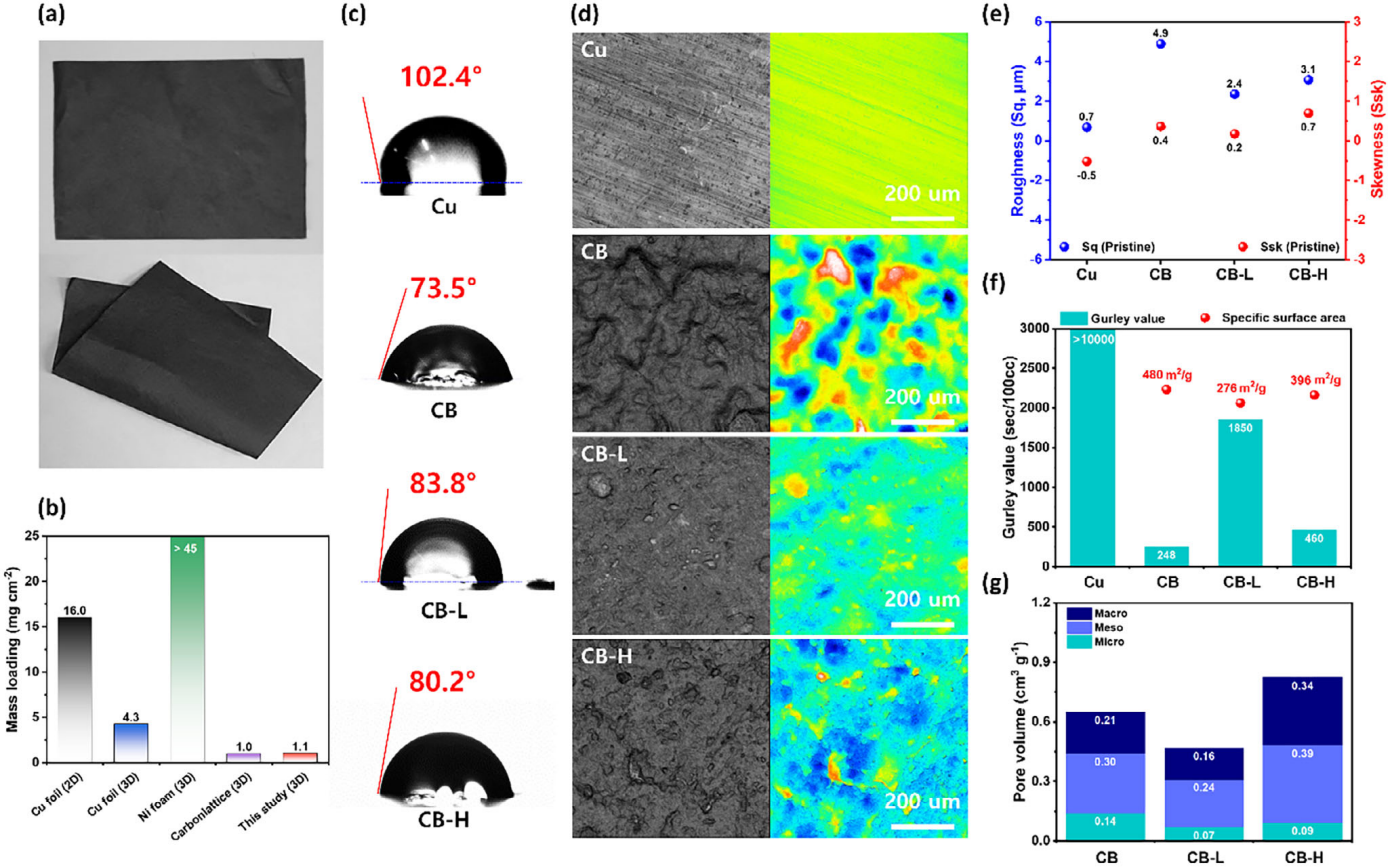
Structural and surface properties of the CBCCs incorporating *d*‐CB, compared with those of the pristine CBCCs and conventional metal CCs. (a) Photographic images of the freestanding CBCC films. (b) Comparison of the areal mass loading between two‐dimensional/3D metal CCs and CBCCs. (c) Water contact angles, showing differences in the surface wettability. (d, e) 3D Topography imaging and measurement of the roughness (Sq) and skewness (Ssk) obtained by CLSM. (f) Gurley air‐permeability values and BET specific surface areas. (g) Pore‐volume distributions partitioned into micro‐, meso‐, and macropores based on the N_2_ adsorption results.

Figure 2 shows the structural and surface property characterization results for lightweight *d*‐CB base current collectors (*d*‐CBCC) assembled from pristine CB and the *d*‐CBs. Specifically, Figure 2a provides photographic images of the freestanding CBCC, demonstrating its excellent flexibility and mechanical resilience. A bending test and stress–strain analysis were performed to confirm its structural stability (Figures S3 and S4). The *d*‐CBCC sample maintained a tensile strength of approximately 14–15 MPa while exhibiting an elongation at break of up to 4.5%, which may contribute to buffering the mechanical stress induced during the lithium plating/stripping process. Additionally, the thickness and mass measurements for the various specimens are listed in Table S1, wherein it can be seen that all samples achieve low areal mass loadings (0.9–1.2 mg for a 12 mm punched disk) and sub‐10 µm thicknesses (7–10 µm), while the Cu foil exhibits corresponding values of 18.2 mg and 18 µm, respectively. Furthermore, the physical advantages of the *d*‐CBCCs are clearly confirmed in Figure 2b, where a comparison is performed with the properties of conventional metal‐based CCs. The areal mass loading data indicate that the *d*‐CBCCs exhibit lower masses per area than the metal‐based collectors, a result that can be attributed to the hierarchically porous structure generated through steam activation. This substantial density reduction (1.1–1.5 g cm^−3^ for the *d*‐CBCCs compared with 8.9 g cm^−3^ for the Cu foil) directly enhances the gravimetric energy density of the resulting batteries due to the presence of a lower fraction of inactive mass within the cell. Figure 2c provides the obtained contact angle images for evaluation of the surface wettability, wherein the enhanced hydrophilicity exhibited by the *d*‐CBCCs suggests that electrolyte access is facilitated compared with the case of the Cu CC. Consequently, uniform Li^+^ flux and suppressed dendrite growth could be achieved in these systems [30]. The 3D surface topography maps presented in Figure 2d and the quantitative roughness (Sq) and skewness (Ssk) data given in Figure 2e are derived from the results of confocal laser scanning microscopy (CLSM). Both measurement parameters follow a comparable trend, with the pristine CB exhibiting the highest values, the defect‐induced samples showing stabilized behavior, and the Cu foil recording the lowest surface roughness along with smooth metrics (Figure S5). That can enhance the density of Li nucleation sites and facilitate a more uniform distribution of the local current during Li plating, thereby limiting the formation of sharp dendrites [31]. Figure 2f provides a comparison of the air permeability characteristics and specific surface areas for the tested samples. As shown, the *d*‐CB powder base sheets (CB‐L and CB‐H) exhibit higher air permeabilities with substantially larger specific surface areas compared with those of the pristine CB sheet. Importantly, these properties facilitate ion and electrolyte transport during electrical testing. Detailed Brunauer–Emmett–Teller (BET) profiles are provided in Figure S6a, wherein a slightly vertical increase is observed for the N_2_ adsorption capacity at lower relative pressures (P/P_0_ ≤ 0.01), corresponding to a Type I isotherm. This result indicates the presence of micropores in the d‐CB sheets, which possess a more developed microporous structure than the pristine CB sample. Details regarding the pore‐volume distributions are presented in Figure 2g, while comprehensive profiles are provided in Figure S6b. From the obtained results, it is apparent that following steam activation, the *d*‐CBCCs exhibit a well‐balanced distribution of hierarchical pores, optimizing both capillary‐driven wetting and Li^+^ ion conduction within the collector framework [32].

**FIGURE 3:**
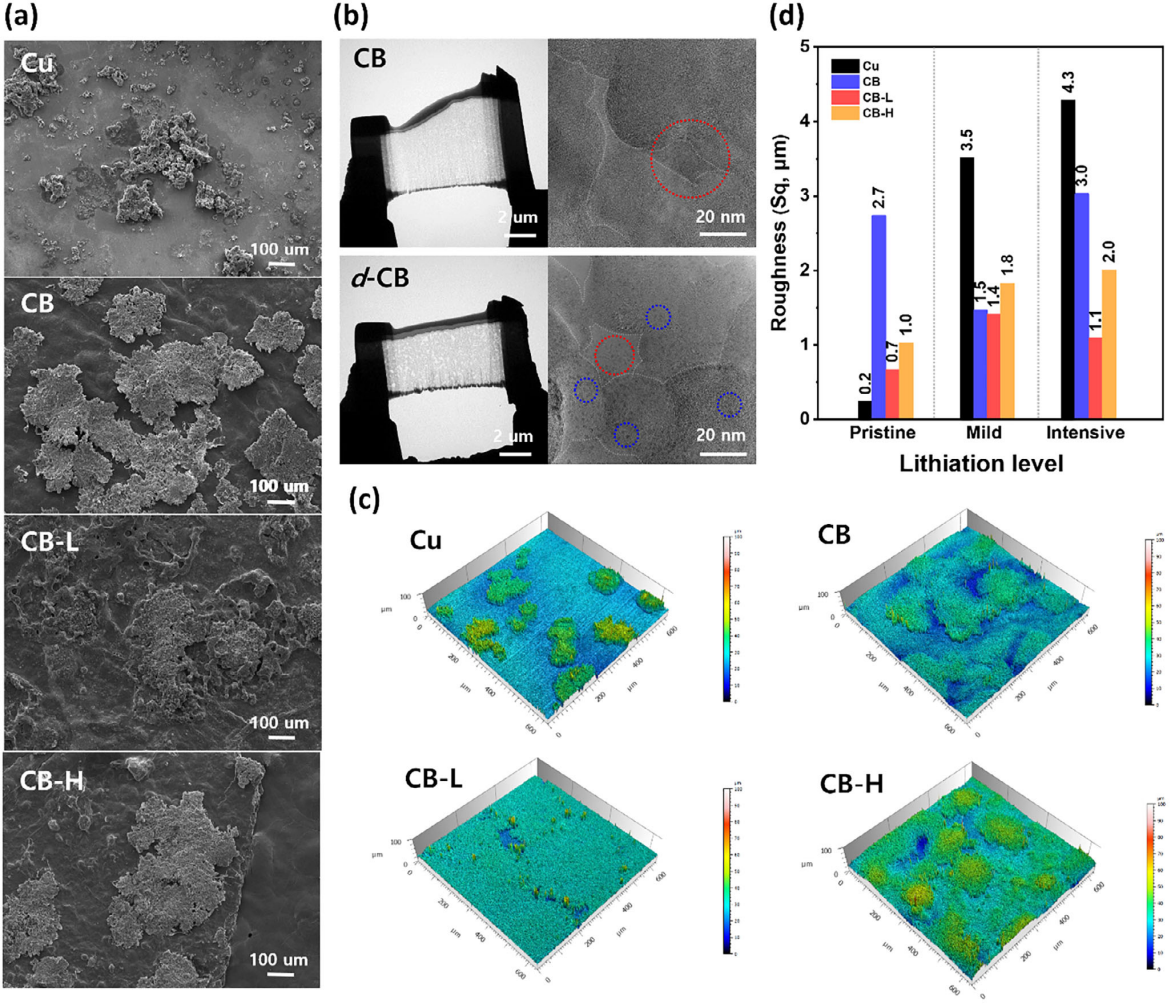
Comparison of the morphology and surface topography of the Li‐plated CBCCs and the pristine Cu foil. (a) Surface FE‐SEM images recorded after Li deposition at a current density of 0.2 mA cm^−2^ until reaching a capacity of 4 mAh cm^−2^. (b) Cs‐TEM images comparing samples without and with defect induction: left, an FIB‐prepared lamella extracted from a CBCC region; right, a high‐magnification view highlighting the Li growth sites. (c) 3D surface maps and (d) roughness (Sq) values obtained from CLSM analysis under different plating levels (mild; 0.2 mA cm^−2^ until reaching 4 mAh cm^−2^, intensive; 0.5 mA cm^−2^ until reaching 10 mAh cm^−2^).

Subsequently, the impact of the lithiation capability was evaluated with respect to the uniformity of Li deposition on the various CCs (Figure 3), wherein the deposition process was controlled by limiting the areal discharge capacity of the cells. Figure S7 shows photographic images of the Li‐plated electrodes after deposition at different current densities. From these images, it is clear that Li deposition on the pristine Cu results in a non‐uniform surface morphology, while the *d*‐CBCC electrodes show more homogeneous and compact Li deposition. At a high areal capacity of 10 mAh cm^−2^, the *d*‐CBCC electrodes retain a more stable surface structure, with the CB‐L specimen exhibiting a highly uniform distribution. Figure 3a shows the corresponding surface field emission scanning electron microscopy (FE‐SEM) images, which compare Li deposition on the Cu foil and the *d*‐CBCCs at a current density of 0.2 mA cm^−2^ up to an areal capacity of 4 mAh cm^−2^. The images clearly reveal morphological differences in the dendritic Li structures, wherein irregular deposition is predominantly observed on the Cu foil surface, with coarse dendrites and significant surface roughness. In contrast, Li deposition on the *d*‐CBCCs, particularly in the cases of the defect‐induced samples (CB‐L and CB‐H), is highly uniform, generating a well‐adhered interface with the surface. To provide a more comprehensive understanding of the plating behavior, Figure S8 presents low‐ and high‐magnification SEM images of Li deposits. The Cu foil exhibits a non‐uniform surface with protruding Li agglomerates, whereas the d‐CBCC electrodes (CB‐L and CB‐H) show a flatter and more homogeneous morphology, indicating lateral Li spreading within the carbon framework. In addition, the size of the deposited Li features was qualitatively evaluated from high‐magnification SEM images, revealing smaller and more uniformly distributed Li domains for the d‐CBCC electrodes compared to the Cu foil, although precise quantification is limited due to the irregular morphology of the deposits. This behavior is further clarified by cross‐sectional FIB‐SEM images (Figure S9), which reveal that Li in the d‐CBCC electrodes penetrates across the scaffold thickness and occupies the internal pore network, rather than forming surface‐confined deposits as observed on the Cu foil. Accordingly, Li is more uniformly distributed across the electrode thickness in the d‐CBCC structure, whereas the Cu electrode shows strong surface localization. High‐magnification images further confirm that CB‐L maintains a more compact and uniform interface than pristine CB, suggesting that defect‐induced sites act as effective nucleation centers that regulate Li growth [13].

While pore‐confined lithium deposition has been reported in various carbon‐based hosts, the present study emphasizes the role of defect‐engineered carbon black in influencing lithium nucleation behavior within a hierarchical porous framework. The introduced defect sites are considered to act as preferential nucleation points, leading to increased nucleation density and more spatially uniform lithium deposition. As a result, lithium growth becomes more evenly distributed throughout the 3D structure, thereby suppressing localized accumulation and irregular protrusion.

The voltage profiles confirm differences in the dendritic Li morphologies between the Cu foil and the *d*‐CBCC specimens. In the defect‐induced CB‐L and CB‐H samples, micrometer‐scale Li deposits can be clearly observed, whereas larger deposits are evident on the pristine CB, and the Cu foil exhibits deposits consistent with irregularly structured bulk Li. Furthermore, high‐resolution Cs‐TEM images of the Li‐plated CB and *d*‐CB specimens are presented in Figure 3b. These images were obtained via cross‐sectional sampling using a focused ion beam to confirm the internal structure, indicating that CB exhibits partial Li plating attributed to the cage effect. In contrast, the *d*‐CB specimen shows Li plating not only within the cage structure, but also on the particle surfaces. This demonstrates that the induced defects serve as Li growth sites. Consequently, the defect‐induced samples demonstrate enhanced internal Li nucleation, directly correlating with the enhanced defect density introduced through steam activation. Accordingly, Li preferentially infiltrates the internal porous framework, thereby effectively minimizing surface protrusions and suppressing dendritic growth. Figure 3c shows the 3D surface maps obtained through CLSM to confirm the differences in surface roughness (Sq) between the Cu foil and the *d*‐CBCCs with mild (0.2 mA cm^−2^, 4 mAh cm^−2^) and intensive (0.5 mA cm^−2^, 10 mAh cm^−2^) plating levels. The quantitative comparison of roughness presented in Figure 3d demonstrates that the *d*‐CBCCs exhibit significantly lower surface roughness than the Cu foil under both plating conditions. These observations are further supported by 3D CLSM images and corresponding line profiles in Figure S11, which confirm the topographical stability of the Li‐plated electrodes. Even under high‐capacity plating conditions, the d‐CBCC electrodes effectively mitigate dendritic growth, resulting in smooth and homogeneous Li deposition. The height profiles clearly demonstrate that surface fluctuations are significantly suppressed in the *d*‐CBCC electrodes compared to the erratic protrusions observed on the Cu foil, as well as pristine CB used as a control reference. Collectively, these comprehensive morphological and topographical data indicate that the hierarchical porous structure of the *d*‐CBCC electrodes provides a stable 3D host environment that regulates Li nucleation and growth. This structural advantage contributes to the formation of a smoother, dendrite‐suppressed interface even under aggressive electrochemical conditions, thereby enhancing the performance and safety of Li‐metal batteries.

**FIGURE 4:**
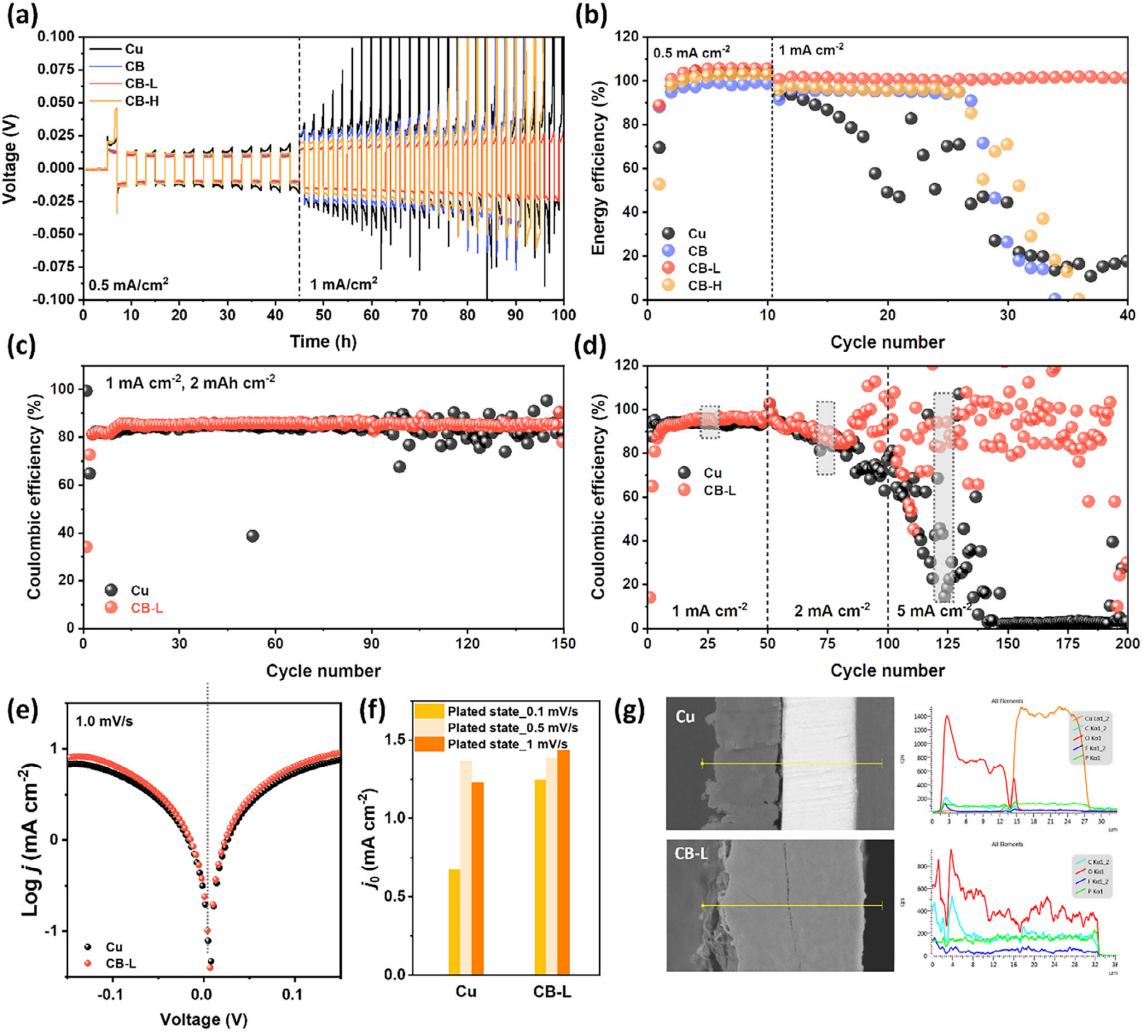
Electrochemical characterization and Li^+^‐transport kinetics for the different CCs (Cu foil, CB, CB‐L, and CB‐H). (a) Galvanostatic plating/stripping profiles and (b) energy efficiencies of the symmetric cells fabricated using the Li CCs. Testing was performed at 0.5 and 1 mA cm^−2^ with a capacity cut‐off of 1.0 mAh cm^−2^. (c) Cycle performances of the Li metal cells incorporating the Cu and CB‐L CCs at 1 mA cm^−2^ with a capacity of 2 mAh cm^−2^. (d) Cycle performances at current densities of 1, 2, and 5 mA cm^−2^ with a capacity cut‐off of 2 mAh cm^−2^. Linear sweep voltammetry of the Li metal//Li CC cells performed between −0.2 and 0.2 V. (e) Tafel plots observed at a scan rate of 1 mV s^−1^ between −0.2 and 0.2 V. (f) Exchange current densities (*i*_0_​) extracted from the Tafel slopes at 0.1, 0.5, and 1 mV s^−1^. (g) Cross‐sectional SEM image and EDS line scan along the yellow lines indicated for the Li‐plated Cu and CB‐L base CCs.

To estimate the Li stability and polarization behavior, Li//CC symmetric cells and Li metal half‐cell tests were conducted, wherein the symmetric cell performance was tested at cycling capacities of 0.5 and 1 mA cm^−2^ with a capacity cut‐off of 1 mAh cm^−2^. The voltage–time profiles presented in Figure 4a show that the CB‐L electrode achieves a lower voltage hysteresis and good lifespan over 400 h compared with the other electrodes, which exhibit fluctuating lifespans of ∼350 h (Figure S12). In particular, the conventional Cu foil shows higher voltage hysteresis than the *d*‐CBCCs, which is attributed to the absence of Li growth sites and the current concentration, likely due to the formation of a flat surface structure [30, 31, 33]. Furthermore, CB‐L exhibits an energy efficiency retention of >99% after 100 cycles, whereas the Cu foil exhibits a gradual decrease and short‐circuit over 80 cycles at 0.5 mA cm^−2^ (Figure S13). Moreover, after 40 cycles under harsh current density conditions, CB‐L achieves stable retention compared with the other electrodes, among which the Cu electrode demonstrates the most drastic reduction (Figure 4b). This indicates that the preferentially modified *d*‐CBCC exhibits superior electrochemical stability compared with the Cu electrode. Upon further comparison of CB‐L with the Cu electrode using the Li‐metal half‐cell configuration (Figure 4c,d), it is apparent that the reversibility of Li plating/stripping in CB‐L is maintained for >150 cycles at 1 mA cm^−2^, while the Cu electrode demonstrates an unstable Coulombic efficiency (CE) after 90 cycles. The CE was further investigated at 2 and 5 mA cm^−2^ (Figure 4d), while the voltage profile behaviors at different current densities are presented for the 25th, 75th, and 125th cycles in Figure S14. At the highest current density (5 mA cm^−2^), the CB‐L and Cu electrodes exhibit CEs of 82% and 21%, respectively. Additionally, the voltage profiles show that CB‐L maintains relatively stable behavior, with a voltage increase of only 217 mV in the 125th cycle. In contrast, Cu displays a voltage increase of 310 mV and greater polarization, indicating that CB‐L promotes more efficient Li deposition with lower resistance (c.f., Cu itself), resulting in higher efficiency and improved cycling stability. The improved performance of CB‐L can be attributed to two key structural features, namely the presence of *d*‐CB and the 3D CNT scaffold. These features enhance Li deposition and minimize irreversible Li loss, ultimately leading to superior cycling stability compared with that of Cu [1, 33].

Figure 4e shows Tafel plots extracted from the Li CC symmetric cells, while Figure 4f provides the corresponding calculated exchange current densities. Figure 4e shows Tafel plots extracted from the Li CC symmetric cells, while Figure 4f provides the corresponding calculated exchange current densities. The higher slope observed for CB‐L reflects reduced interfacial resistance, while the significantly higher exchange current density underscores enhanced Li‐ion kinetics and interfacial charge transfer. To further elucidate the plating mechanism, XRD patterns were analyzed at different capacities (Figure S15). XRD analysis reveals distinct Li deposition behaviors between *d*‐CBCC and Cu electrodes. The Cu electrode exhibits a stronger Li_2_CO_3_ signal, signifying more pronounced parasitic reactions, in good agreement with previous reports. [34, 35] At 4 mAh cm^−^^2^, *d*‐CBCC shows a larger Li (110) peak area, reflecting facilitated Li nucleation within its defect‐rich carbon framework. This behavior arises from defect sites in defect‐engineered carbon hosts that act as energetically favorable nucleation centers for Li deposition [35, 36]. At 10 mAh cm^−^^2^, although the Li (110) peak intensity becomes higher for the Cu electrode, the peak exhibits a clear leftward shift and splitting, indicating lattice distortion and increased structural disorder associated with non‐uniform Li deposition. Notably, the increased peak intensity does not correspond to stable deposition, but rather to the accumulation of electrochemically isolated Li associated with heterogeneous growth [34, 35, 36]. In contrast, *d*‐CBCC maintains a stable peak profile even at higher capacity, indicating more uniform Li deposition within the conductive carbon framework. Overall, the improved kinetics arise from the increased active surface area and optimized architecture of CB‐L, which are associated with the underlying d‐CBCC structure. Figure 4g presents cross‐sectional SEM images and the corresponding EDS elemental mapping images recorded for the CB‐L and Cu electrodes after Li plating up to a capacity cut‐off of 4 mAh cm^−^^2^. These images show that Li growth occurs within the internal CB‐L scaffold rather than forming isolated surface deposits, in contrast to the Cu electrode. EDS mapping further confirms a more uniform distribution of Li within the scaffold, whereas the Cu electrode exhibits surface crowding and dendritic growth. These observations further corroborate the XRD results, confirming that Li deposition is more uniformly accommodated within the d‐CBCC structure. Figure 4g presents cross‐sectional SEM images and the corresponding energy‐dispersive x‐ray spectroscopy (EDS) elemental mapping images recorded for the CB‐L and Cu electrodes after Li plating up to a capacity cut‐off of 4 mAh cm^−2^. These images reveal that Li growth occurs within the internal CB‐L scaffold rather than through the formation of isolated surface deposits (c.f., Cu foil). EDS mapping of Li confirms its uniform distribution within the interior of the scaffold, thereby demonstrating the capability of *d*‐CB to accommodate Li internally. In contrast, in the case of the Cu electrode, Li induces surface crowding and dendrite growth.

**FIGURE 5:**
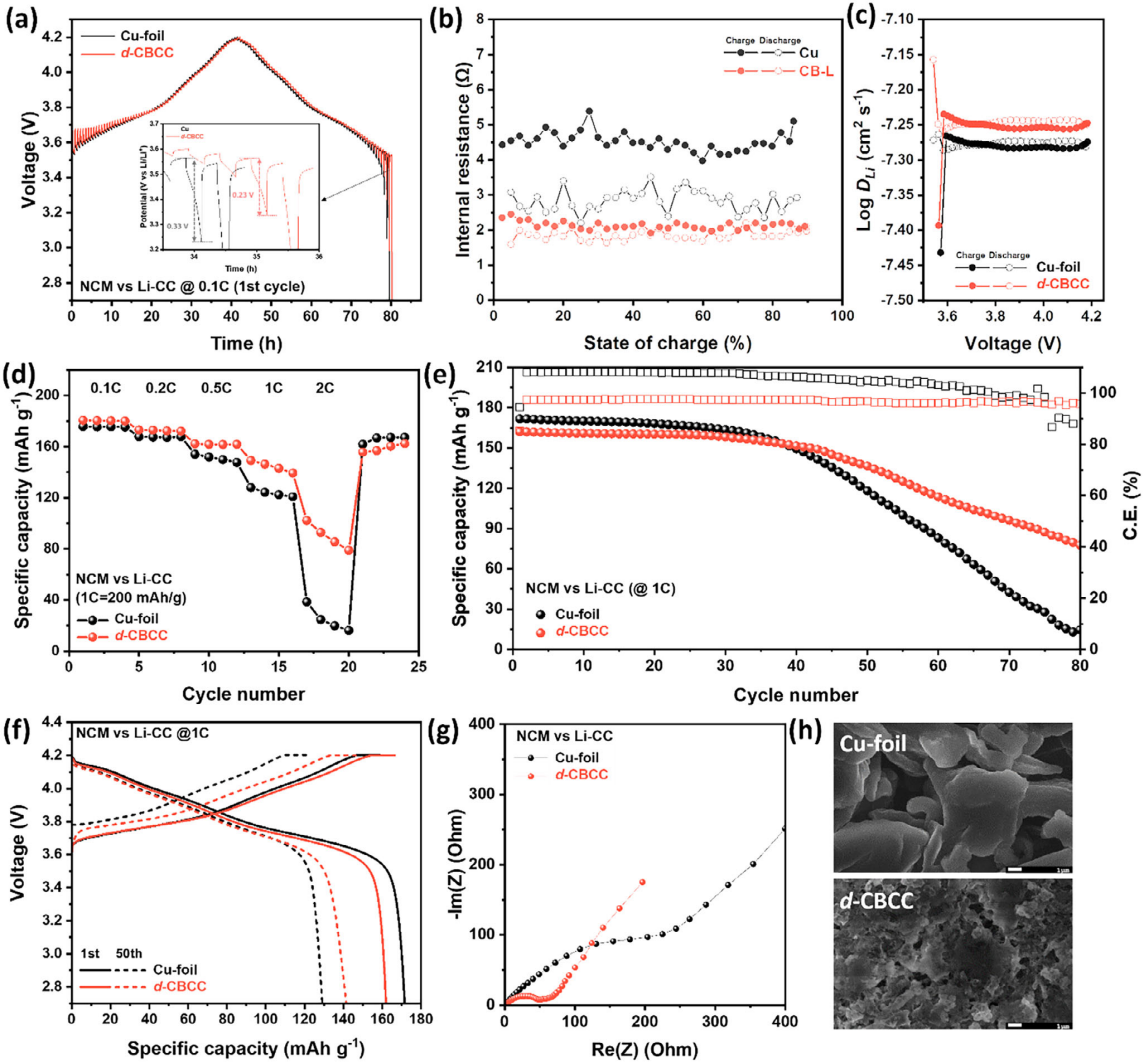
Electrochemical performances of AFLMBs employing *d*‐CB CCs paired with NCM811 cathodes. (a) GITT profiles recorded during the first cycle at 0.1 C. (b) State of charge‐dependent polarization. (c) Apparent Li^+^ diffusion coefficients​ as a function of voltage, as determined from the GITT profiles. (d) Rate capabilities at 0.1–2 C. (e) Capacity retention during cycling at 1 C. (f) Galvanostatic charge–discharge profiles recorded for the first and 50th cycles. (g) Nyquist plots recorded after the cycling test (5 mV, 250 kHz–10 mHz) (h) Surface FE‐SEM images of the anodes after the cycling test.

To verify the practical applicability of the *d*‐CB and Cu‐based CCs in AFLMBs, the performances of full cells were evaluated in conjunction with a layered nickel‐rich LiNi_0.8_Co_0.1_Mn_0.1_O_2_ (NCM811) cathode (Figure 5). Pre‐activation was performed at 0.2 mA cm^−^^2^ for all CCs prior to full‐cell assembly to ensure surface stabilization. The Li^+^ diffusivity at the electrode interface was tracked using the galvanostatic intermittent titration technique (GITT) during the initial cycle, and the corresponding results are presented in Figure 5a–c. Specifically, Figure 5a shows that the GITT profile recorded for *d*‐CB exhibits lower electrochemical polarization (0.23 V) than the Cu electrode (0.33 V) during discharge at 0.1 C (1C = 200 mAh g^−1^). Additionally, the changes in the Li^+^ diffusion coefficient during charge and discharge are shown in Figure 5b. Overall, the *d*‐CB electrode ($LogD_{Li^{+}}\approx -7.39\;to-7.22cm^{2}s^{-1}$) maintains faster reaction kinetics across the entire voltage range compared with the Cu foil ($LogD_{Li^{+}}\approx -7.43\;to-7.27cm^{2}s^{-1}$), which is further supported by the extracted diffusion coefficients presented in Figure S16. This behavior is also associated with the internal resistance variations, wherein the *d*‐CB electrode exhibits more stable and lower resistance than the Cu electrode, as shown in Figure 5b. These features are associated with high‐rate charge‐discharge operations, as quantitatively illustrated in Figure 5d. Specifically, the Cu electrode exhibits a gradual performance degradation at 2C compared to the *d*‐CB electrode, retaining only 24.6% of its capacity at 0.5 C. In contrast, the *d*‐CB electrode maintains 56.5% of its initial capacity, thereby clearly demonstrating its superior rate performance and adaptability to fast‐charging conditions. Furthermore, the CE of the Cu electrode declines beyond the 30th cycle, resulting in a capacity retention of only 9.0% after the 80th cycle. By comparison, the *d*‐CB electrode shows a more gradual decrease, maintaining 50.1% retention and sustaining a stable CE throughout the cycling period. Although Cu electrodes exhibit a relatively high Coulombic efficiency (CE) in the initial cycles, this does not necessarily lead to improved long‐term capacity retention. This can be attributed to the fact that CE primarily reflects the reversibility of lithium plating/stripping in each cycle and does not adequately capture cumulative lithium loss, such as dead Li formation and continuous SEI growth [37, 38]. Dead Li, defined as electrically isolated lithium formed during repeated plating/stripping, is widely recognized as a dominant contributor to irreversible capacity loss and long‐term cycling instability in lithium metal batteries [11, 12, 13]. In the case of the Cu electrode, non‐uniform Li deposition promotes the formation of dendritic and electrically isolated Li, which accelerates dead Li accumulation during cycling and leads to rapid capacity decay. This interpretation is further supported by our additional XRD and morphology analyses, where the Cu electrode shows stronger Li_2_CO_3_ formation, unstable peak evolution (Figure S15), and non‐uniform Li deposition morphology, all of which are consistent with heterogeneous growth and dead Li accumulation. In contrast, the porous and defect‐rich *d*‐CB framework facilitates more uniform Li nucleation and growth within the conductive matrix, thereby effectively suppressing the formation of dead Li and improving long‐term cycling stability. This suppression of dead Li formation is attributed to the confined Li deposition within the interconnected porous framework, which minimizes electrical isolation and maintains continuous electronic pathways during repeated cycling. To isolate the effect of the CNT framework from that of defect‐engineered carbon black, the cycling performance is further compared between 3D‐CNT and *d*‐CB, and between CB and *d*‐CB current collectors (Figures S17 and S18, respectively).

The long‐term capacity retention of CB and 3D‐CNT is similar, whereas *d*‐CB maintains substantially improved retention, underscoring the dominant role of defect‐induced lithiophilicity in stabilizing cycling behavior. Figure 5f displays the voltage profiles recorded over these extended cycles, revealing minimal capacity fading and low polarization buildup. These results indicate that the *d*‐CB CC not only supports reversible Li deposition, but that it also stabilizes the electrode–electrolyte interface during prolonged use. Additionally, to verify its compatibility with various cathode systems, full‐cell tests were conducted utilizing a lithium iron phosphate (LFP) cathode. These tests further demonstrated that the modified current collector maintained lower polarization and improved reversibility after cycling (Figure S19). Furthermore, to substantiate the robustness of the *d*‐CBCC architecture across different chemical environments, additional evaluations were conducted using an ether‐based electrolyte (1 m LiTFSI in DOL/DME with 2 wt.% LiNO_3_). The *d*‐CBCC retained 81.1% of its initial capacity after 70 cycles at 1C, whereas the Cu foil exhibited a rapid decay to only 8.2% (Figure S20). This confirms that the structural benefits of *d*‐CBCC are consistently maintained regardless of the electrolyte system. The enhanced interfacial stability of the *d*‐CB electrode is further corroborated by the electrochemical impedance spectroscopy (EIS) results presented in Figures 5g and Figure S21. Before cycling, the charge‐transfer resistances (*R*_ct_) of the Cu and *d*‐CB electrodes were determined to be 14.7 and 23.3 Ω, respectively. The slightly higher *R*_ct_ observed at the *d*‐CB base system can be attributed to its more porous and defect‐rich carbon surface relative to that of the metallic Cu, which requires a longer interphase stabilization time. After cycling, the Nyquist plot for *d*‐CB shows only a modest increase in resistance (*R*_ct_ = 56.2 Ω), whereas that of Cu (*R*_ct_ = 322.5 Ω) exhibits a pronounced increase. This indicates superior interfacial stability for the *d*‐CB electrode, consistent with the formation of a stable solid–electrolyte interphase and an efficient charge transfer ability. The FE‐SEM images presented in Figure 5h depict the Li‐deposition morphology following extended cycling. As shown, the *d*‐CB sample exhibits a smooth, compact Li layer characterized by minimal dendritic features, in contrast to the conventional Cu electrode, which displays a rough, uneven morphology with pronounced dendritic growth. These observations indicate that the *d*‐CB base current collector effectively regulates Li plating via its porous, defect‐rich architecture, facilitating uniform deposition within the collector matrix rather than on the surface. Collectively, these results establish the practical viability of modified *d*‐CBCCs for use in AFLMBs, as they promote uniform Li plating, maintain high‐rate performances, and provide extended cycle lives, thereby positioning these materials as credible alternatives to traditional metallic CCs in next‐generation high‐energy systems.

## 3 Conclusion

In this study, a hierarchically porous, architecture‐controlled *d*‐CBCC was developed by integrating steam‐activated carbon black with carbon nanotubes. This binder‐free, lightweight, and mechanically robust scaffold was enriched with defect sites introduced by steam activation, which provided abundant lithiophilic nucleation centers while preserving graphitic conductivity. These architectural features facilitated uniform Li growth within the internal porous network, thereby suppressing dendritic formation and mitigating interfacial instabilities at the Li‐metal anode. Electrochemical analyses demonstrated that the prepared *d*‐CBCCs, particularly the defect‐engineered variant with a low defect density, achieved high Coulombic efficiency, stable cycling performance, and superior rate capability compared with those exhibited by conventional Cu foil. When paired with a high‐nickel NCM811 cathode in an anode‐free full‐cell configuration, the optimal *d*‐CBCC enabled reversible Li plating/stripping, retained 82.8% of its initial capacity over 50 cycles, and maintained a smooth Li morphology without dendrite formation. In contrast to conventional chemical modification strategies that frequently compromise the electrical and mechanical properties of carbon frameworks, this steam activation method introduces lithiophilic defects through a physical process. This approach preserves graphitic conductivity and maintains the integrity of the ultralight carbon nanotube‐supported scaffold, thereby providing a scalable and industry‐compatible solution for advanced architecture‐controlled current collectors. By combining ultralight mass loading with enhanced Li affinity, high electronic conductivity, and structural robustness, the proposed *d*‐CBCC provides a practical pathway toward next‐generation Li metal batteries, particularly for applications requiring high energy density and stringent weight and safety constraints, such as aerospace, defense systems, advanced portable electronics, and next‐generation electric mobility.

## 4 Experimental Section

### 4.1 Preparation of the Defect‐Induced Carbon Black Powder

Commercial acetylene black (Denka Black Li‐435, Denka Korea Co., Ltd.) was used as the pristine CB for the purpose of this study. To introduce structural defects into the CB while minimizing excessive damage, *d*‐CB was obtained by steam activation under different conditions in a tube furnace. The temperature was increased 25°C to 950°C at a rate of 10°C min^−^^1^ in an N_2_ atmosphere (200 mL min^−1^), and steam (400 mL min^−1^) was introduced as the activating agent using air as the carrier gas. Activation was performed for 15 min (CB‐L) or 60 min (CB‐H). The designations “L” and “H” denote low and high defect densities induced via steam activation, respectively.

### 4.2 Preparation of the 3D Carbon Black‐Based CC Anodes

The single‐walled carbon nanotube (SWCNT) powder and the prepared CB samples (CB, CB‐L, and CB‐H) were mixed in a 1:1 weight ratio at 500 rpm for 5 min (Thinky mixer). The resulting mixture was then dispersed in deionized water under ultrasonication conditions (250 W, 2 h, 15°C), prior to vacuum‐filtration through qualitative filter paper (No. 53, Hyundai Micro Co., Ltd.). The obtained solids were dried in a vacuum oven at 120°C for 24 h, then carefully detached from the filter paper to obtain free‐standing carbon papers that were used as the anode side current collectors in the cell configuration.

### 4.3 Preparation of the Commercial Cathode

The cathode was prepared using NCM811 (Ecopro, South Korea) and LFP (Ecopro, South Korea) as the active material, Super‐P black as the conducting material, and polyvinylidene fluoride dissolved in N‐methyl‐2‐pyrrolidone (12 wt.%, Kureha Co, Ltd.) as the binder, with a corresponding weight ratio of 96:2:2. The mixed slurry was coated onto an Al foil and dried at 80°C for 24 h to produce the cathode material for incorporation into the anode‐free cells.

### 4.4 Characterization

The surface morphologies of the samples were analyzed by FE‐SEM (JEOL, JSM‐6700F, Japan) and 3D CLSM (Carl Zeiss, LSM 800 MAT, Germany). Cs‐TEM (JEM‐ARM200F, JEOL, Japan) was used to investigate the morphologies and crystallinities of the prepared powders and electrodes. The crystalline properties were investigated using XRD (Rigaku D/Max 2500/PC, Japan), and the existence of disordered carbon structures was confirmed by Raman spectroscopy (HORIBA Jobin Yvon, LabRAM ARAMIS, Japan) using an Ar‐ion laser beam. The elements present in the prepared specimens were identified using XPS (PHI 5000 VersaProbe, ULVAC‐PHI Inc., Osaka, Japan). Additionally, the surface areas and pore characteristics of the samples were determined using a gas analyzer (BELSORP‐max, MicrotracBEL Corp., Japan). BET and NLDFT models were used to calculate the specific surface areas and micropore‐ and mesopore distributions of the samples from their corresponding N_2_ adsorption–desorption isotherms.

### 4.5 Electrochemical Measurements

The electrochemical stabilities of Li deposition and dissolution were assessed in Li metal cells incorporating either bare Cu foil or the prepared CBCCs. Li metal foil was physically affixed to a stainless steel spacer (1T) and used as the counter electrode, with a polyolefin membrane (Toray) serving as the separator. The full cell configuration consisted of commercial NCM811 as the cathode and the desired CBCC as the anode. Both electrodes were fabricated into 12 mm disks for experimental use. A 1 m lithium hexafluorophosphate solution was used as the electrolyte in a mixture of ethylene carbonate, dimethyl carbonate, and ethyl methyl carbonate (2:4:4, a volume ratio), supplemented with 12.5% fluoroethylene carbonate. All cell tests are conducted at 25°C in a constant temperature chamber.

## Author Contributions

**Kwang Chul Roh**: resources, supervision, and funding acquisition. **Dong Hyeon Hwa**: data curation, methodology. **Ji Su Chae**: supervision, funding acquisition, data curation, writing – review and editing, investigation. **Seo Hui Kang**: conceptualization, methodology, data curation, investigation, validation, formal analysis, visualization, writing – original draft, writing – review and editing.

## Conflicts of Interest

The authors declare no conflicts of interest.

## Supporting information




**Supporting File**: advs75769‐sup‐0001‐SuppMat.docx.

## Data Availability

The data that support the findings of this study are available from the corresponding author upon reasonable request
