# Supplementary material for: Architecture‐Controlled Hierarchical Carbon‐Based Current Collector for Mitigating Interfacial Instabilities in Lithium‐Metal Anodes
Source: Adv Sci (Weinh). 2026 May 22;13(46):e75769. doi: 10.1002/advs.75769 (PMC13335991; doi:10.1002/advs.75769)
Supplement: Supplementary file 1 [file ADVS-13-e75769-s001.docx]

Supporting Information

Architecture-Controlled Hierarchical Carbon-Based Current Collector for Mitigating Interfacial Instabilities in Lithium-Metal Anodes

Seo Hui Kang^1, 2^, Dong Hyeon Hwa^1, 3^, Ji Su Chae*^,1^, and Kwang Chul Roh*^,1^

S. H. Kang, D. H. Hwa, J. S. Chae, K. C. Roh

Climate and Energy Research Group, Korea Institute of Ceramic Engineering and Technology, Jinju, Gyeongsangnam‑do 52851, Republic of Korea

E-mail: rkc@kicet.re.kr, jschae0@kicet.re.kr

S. H. Kang

Space & Energy Technology Team, Innovation Technology Research Division, Korea Research institute for defense Technology planning and advancement, Daejeon 35287, Republic of Korea

D. H. Hwa

Department of Ceramic Engineering, School of Materials Science and Engineering, College of Engineering, Gyeongsang National University, Jinju, Gyeongsangnam‑do 52851, Republic of Korea

**Figure S1.** Deconvoluted Raman spectra of CB, CB-L, and CB-H powders, showing the evolution of D, G, D′, 2D, and D+D′ bands with increasing activation.

**Figure S2.** X-ray diffraction patterns of pristine CB and d-CBs (CB-L, CB-H) powders.

**Figure S3.** Photographs of freestanding carbon-based current-collectors: top, thickness measurements; bottom, bending test demonstrating mechanical robustness.

**Figure S4.** Tensile stress–strain curves of carbon-based current collectors

**Table S1.** Physical parameters of Cu and carbon-based current collectors

| Current collector | Cu | CB | CB-:L | CB-H |
| --- | --- | --- | --- | --- |
| Mass (mg)_a) re collectors. cycle. (d) CE | 18.2 | 0.9 | 1.2 | 1.2 |
| Thickness (um) | 18.0 | 7.0 | 7.0 | 10.0 |
| Density (g cc^-2^) | 8.9 | 1.1 | 1.5 | 1.1 |

* All samples were punched to a diameter of 12π um.

**Figure S5.** Extracted 2D roughness profiles (ISO 4287) showing amplitude and material ratio parameters along the horizontal line from the CLSM image.

**Figure S6.** (a) Nitrogen adsorption–desorption isotherms of CNT, CB, CB-L, and CB-H. (b) Pore size distributions of the corresponding samples calculated by the NLDFT method.

**Figure S7.** Photographs of Li-plated electrodes after deposition at 0.2 mA cm^−2^ to 4 and 10 mAh cm^−2^ on bare Cu and CBCCs, respectively

**Figure S8.** FE-SEM images at various magnifications of Li deposits formed on bare Cu foil and d-CBCCs after plating at 0.2 mA cm^-2^ to an areal capacity of 4 mAh cm^-2^ .

**Figure S9.** Cross-sectional FE-SEM images of Cu and d-CBCCs obtained via FIB milling after Li plating at 0.2 mA cm^-2^ to an areal capacity of 4 mAh cm^-2^ .

**Figure S10.** Voltage profiles of Li-metal//CC cells at 0.2 mA cm^−2^ to 4 mAh cm^−2^.

**Figure S11.** 3D topography imaging and surface height profiles of Cu foil and d-CBCCs obtained via CLSM analysis after Li-plating under mild (0.2 mA cm⁻² to 4 mAh cm⁻²) and intensive (0.5 mA cm⁻² to 10 mAh cm⁻²) conditions.

**Figure S12.** Galvanostatic plating/stripping profiles of symmetric Li-CC cells

at 0.5 mA cm⁻^2^ (capacity cut-off = 1 mAh cm⁻^2^).

**Figure S13.** Cycle retention of symmetric cell at 0.5 mA cm⁻^2^ (capacity cut-off = 1 mAh cm⁻^2^).

**Figure S14.** Galvanostatic charge–discharge profiles of symmetric Li//Li-CC cells at 1, 2, and 5 mA cm⁻^2^ (capacity cut-off = 1 mAh cm⁻^2^).

**Figure S15.** XRD patterns of Li deposited on d-CBCC and Cu electrodes at 4 and 10 mAh cm⁻². The Cu electrode shows stronger Li₂CO₃ signals and peak shift and splitting at high capacity, indicating increased structural disorder, while *d*-CBCC maintains a relatively stable peak profile.

.

**Figure S16.** Voltage-dependent Li⁺ diffusion coefficients of Cu and CB-L current collectors extract from GITT test.

**Figure S17.** Electrochemical performances of NCM811 full cells employing 3D-CNT scaffold and d-CBCC current collectors: (a) capacity retention over 80 cycles at 1C and (b) galvanostatic charge–discharge profiles at the 1st and 50th cycles.

**Figure S18.** Electrochemical performances of NCM811 full cells employing CB and d-CB current collectors: (a) capacity retention over 80 cycles at 1C and (b) galvanostatic charge–discharge profiles at the 1st and 50th cycles.

.

**Figure S19.** Galvanostatic charge–discharge profiles of Cu-foil and *d*-CBCC based LFP full cells for the 1st and 50th cycles.

**Figure S20.** Cycling performance of LFP full cells using Cu foil and d-CBCC in a LiTFSI/DOL/DME electrolyte with LiNO₃ additive at 1 C.

**Figure S21.** Nyquist plots of Cu-foil and *d*-CBCC cells at open-circuit voltage before cycling (inset; enlarged high-frequency region).
